# Childhood events as factors in continued cannabis use in adulthood: a longitudinal study of a 30-year follow-up cohort

**DOI:** 10.1186/s42238-025-00345-0

**Published:** 2025-11-12

**Authors:** Solène Wallez, Filiz Eren, Isabelle Kousignian, Guillaume Avenin, Maria Melchior, Murielle Mary-Krause

**Affiliations:** 1 Sorbonne Université, INSERM,, Institut Pierre Louis d’Epidémiologie et de Santé Publique, IPLESP, Equipe en Epidémiologie Sociale, Santé Mentale et Addictions, ESSMA, Paris, F75012 France; 2 BioSTM « Biostatistique, Traitement et Modélisation des données biologiques », US25 Inserm, UAR 3612 CNRS, Faculté de Pharmacie de Paris, Université Paris Cité, Paris, F-75006 France; 3 Sorbonne Université, Faculté de Santé Saint-Antoine, Département de Médecine Générale, Paris, 75012 France; 4 Sorbonne Université - Faculté de Santé Site Saint-Antoine, UMR-S 1136 – N° BC 2908, 27 rue Chaligny, Paris, 75012 France

**Keywords:** Cannabis, Longitudinal study, Trajectory analysis, Long-term use, Predictors, Associated factors

## Abstract

**Background:**

Cannabis use patterns have evolved over time, with increasing persistence into adulthood. This study aims to identify cannabis use trajectories from adolescence to adulthood and to examine the influence of early individual and family factors on these trajectories.

**Methods:**

This study included 622 participants from the French TEMPO cohort who reported cannabis use between 1999 and 2021, based on 14 measurements points. Cannabis consumption from adolescence to adulthood (ages 15 to 46) was assessed using Group-Based Trajectory Modelling (GBTM). Associations with early individual and family factors were examined using multinomial logistic regression.

**Results:**

We identified three distinct cannabis use trajectories: declining consumption (69.9%), fluctuating consumption (13.7%), characterized by an initial increase followed by a decrease, and persistent consumption (16.4%). The fluctuating use trajectory was associated with being male (OR = 2.15, 95%CI = 1.31–3.54), having parents who smoked (OR = 2.18 95%CI = 1.18–4.02), and experiencing parental conflict, stress, or frequent absence before age 17 (OR = 1.93, 95%CI = 1.15–3.23). The persistent use trajectory was associated with being male (OR = 3.66, 95%CI = 2.19–6.09), academic difficulties (OR = 2.47, 95%CI = 1.45–4.22), and early initiation of cannabis (OR = 2.31, 5%CI = 1.11–4.79) or both tobacco and cannabis (OR = 3.07, 95%CI = 1.57–6.02).

**Conclusions:**

These findings underscore the importance of early prevention and intervention strategies, particularly in populations where cannabis use persists despite legal restrictions. Given the potential health and social consequences of prolonged cannabis use, it is essential to identify early-life risk factors to inform targeted policy measures aimed at reducing consumption and preventing cannabis use disorders.

**Supplementary Information:**

The online version contains supplementary material available at 10.1186/s42238-025-00345-0.

## Introduction

Cannabis remains a global concern and is the most widely used illicit drug worldwide. Over the past 10 years, the number of people using cannabis has increased by 34% (United Nations Office on Drugs and Crime, (United Nations Office on Drugs and Crime 2025)). In some countries, such as the United States, prevalence rates have doubled over the past decade, from 12.6% in 2011 to 24.9% in 2021 for past-year cannabis use among adults aged 35–50, and from 26.7% to 42.6% among those aged 19–30 (Butler Center for Research 2023).

In Europe, the situation is also notable. In 2025, 8.4% of European adults (24 million people aged 15–64) reported cannabis use in the past year (European Union Drugs Agency 2025). Importantly, cannabis use is significantly more prevalent among younger populations. Among Europeans aged 15–24, 18.6% reported cannabis use in the past year, and 10.1% in the past month (European Union Drugs Agency (European 2025)).

In 2015, 16-year-olds in France had one of the highest rates of experimentation in Europe (31%), whereas today, with a rate of experimentation of around 8% in 2024 vs. a European mean of 12.0%, they are among the young Europeans least affected by its use (Spilka et al. 2025). Nevertheless, even decreasing, the prevalence of cannabis use is still high in France among young people, with 16.2% of 10th grade students having consumed it in 2022, and 31.2% for 12th grade (Unité data, (Unité Data 2024)). In this country, cannabis use among adults has been rising (Observatoire français des drogues et des tendances addictives 2019), with daily use increasing from 1.4% in 2014 to 2.0% in 2017 among 35–44-year-olds, and from 0.6% to 1.2% among 45–54-year-olds (Spilka et al. 2018). This trend suggests that earlier generations of cannabis users have maintained their consumption into later adulthood.

The early use of cannabis in adolescence is particularly concerning due to its potential impact on brain development. Indeed, according to the scientific literature, early onset of substance use can interfere with adolescent brain development, potentialy resulting in both short- and long-term consequences (Jacobus and Tapert 2014; Jacobus et al., 2019; Lorenzetti et al. 2020). Early use has also been shown to be associated with a greater likelihood of progressing to regular use in late adolescence and early adulthood (Millar et al. 2021).

Although 30% of users initiate cannabis use before the age of 14 (Hawke et al. 2020), many individuals spontaneously quit in their twenties (Terry-McElrath et al. 2017; von Sydow et al. 2001). However, for some, cannabis consumption persists into adulthood, increasing the risk of developing lifetime Cannabis Use Disorder, which affects about one in five individuals before the age of 30 (Farmer et al. 2015). Additionally, long-term cannabis consumption can have detrimental effects on physical, psychological, and cognitive health (Ford et al. 2017; Hall and Degenhardt 2014), including dependence and increased risk of using other illicit substances (Boden et al. 2020; Ellickson et al. 2004; Swift et al. 2012; Taylor et al. 2017).

Moreover, the gender gap in cannabis use has been narrowing over time in some countries, with a relatively greater increase in prevalence among women compared to men (Chapman et al. 2017). Among women, specific concerns arise regarding pregnancy and the potential effects of cannabis use on fetal development (Ainiti et al. 2023; Reyentanz et al. 2025).

In terms of socio-economic consequences, long-term cannabis users tend to have lower educational attainment, reduced income levels, and higher unemployment rates (Boden et al. 2020; Brook et al. 2011; Ellickson et al. 2004; Fergusson and Boden 2008; Thompson et al. 2019). Early cannabis users are also three times more likely to drop out of school without a diploma compared to those who begin later (Vergunst et al. 2022), and they face a heightened risk of unemployment (Barry et al. 2022). Mental health conditions such as anxiety and depression (Brook et al. 2011; Caldeira et al. 2012; Hines et al. 2016), as well as physical health issues (Caldeira et al. 2012; Ellickson et al. 2004; Terry-McElrath et al. 2017; Thompson et al. 2019), are also more prevalent among heavy users. Socially, long-term users tend to experience greater relationship difficulties, lower relationship satisfaction (Brook et al. 2011; Fergusson and Boden 2008), decreased marital harmony (Brook et al. 2011), an increased risk of intimate partner violence (Boden et al. 2020), and a lower likelihood of being in a stable relationship (Boden et al. 2020; Brook et al. 2011).

Regarding factors associated with later cannabis use, although all patterns of use, whether heavy, persistent, early, or occasional, are associated with subsequent cannabis-related problems (Swift et al. 2008), two key predictive factors have been consistently identified: early initiation and early regular use (Copeland et al. 2014; Denissoff et al. 2022; Hall 2006; Kalant 2004; Silins et al. 2014; Perkonigg et al. 1999).

Several childhood factors have been associated with cannabis use, including adverse childhood experiences (Boden et al. 2020; Kosty et al. 2017), parental substance use, family instability, and exposure to sexual abuse (Boden et al. 2020). Cannabis use trajectories have also been linked to aggressive behavior (Brook et al. 2011), and criminal activity (Passarotti et al. 2015) in adolescence. Furthermore, having a partner or peers who use cannabis increases the likelihood of use during adolescence and young adulthood (Brook et al. 2011; Passarotti et al. 2015).

To better understand the various trajectories of cannabis use, it is essential to examine a broad range of contributing factors. Although some studies have investigated long-term patterns of cannabis use (Boden et al. 2020; Brook et al. 2011; Epstein et al. 2015; Hines et al. 2023; Leadbeater et al. 2022; Marmet et al. 2021; Passarotti et al. 2015; Pollard et al. 2018; Terry-McElrath et al. 2017; Vergunst et al. 2022; Windle et al., 2004), their limitations in terms of generalizability, scope, or methodological design underscore the need for current research. Many focus on specific populations, such as men (Marmet et al. 2021; Vergunst et al. 2022), or individuals born in a particular location and year (Boden et al. 2020), while others fail to control for family substance use (Brook et al. 2011; Passarotti et al. 2015; Pollard et al. 2018; Terry-McElrath et al. 2017) or adverse childhood events (Terry-McElrath et al. 2017). Most followed adolescents into young adulthood but not into later adulthood (Epstein et al. 2015; Hines et al. 2023; Leadbeater et al. 2022; Passarotti et al. 2015; Pollard et al. 2018; Windle et al., 2004), a period during which cannabis use can evolve and for which associated factors may differ from those predicting cannabis use in young adults. Moreover, the majority of studies have been conducted in the United States (Brook et al. 2011; Epstein et al. 2015; Passarotti et al. 2015; Pollard et al. 2018; Terry-McElrath et al. 2017; Windle et al., 2004) or other Anglophone countries (Boden et al. 2020; Hines et al. 2023; Leadbeater et al. 2022), and cannabis use patterns may vary across cultural contexts. The literature have highlighted that cannabis use is interwoven with, and influenced by, social, legal, economic, and cultural environments, which often differ across countries and cultures (Cousijn et al. 2024; Rafei et al. 2023; Siddiqui et al. 2022). These differences may contribute to variations in norms and risk perceptions related to cannabis use (Cui et al. 2023; Wu et al. 2015).

Therefore, the aim of this study is to identify cannabis use patterns from adolescence to adulthood and to determine the childhood and adolescent factors that predict long-term consumption, with the hypothesis that adverse childhood events have influence cannabis use in adulthood and that risks factors identified in Anglophone countries also characterize the French population.

## Materials and methods

### Study design and participants

**Fig. 1:**
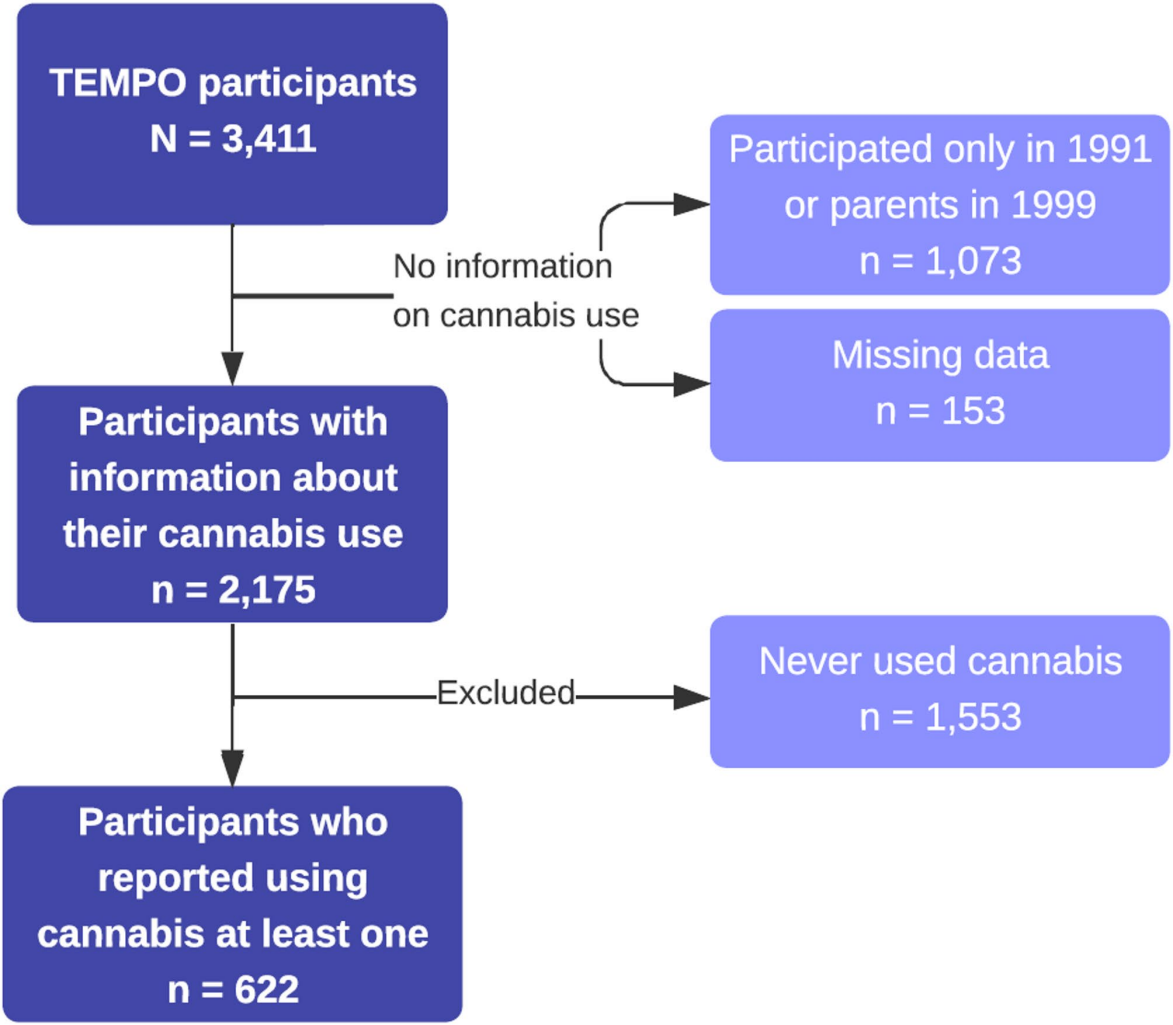
Flow-chart of the study

We analyzed data from the French longitudinal TEMPO cohort (Mary-Krause et al. 2021), initially recruited in 1991 from the offspring of GAZEL participants (one child per family, aged 4 to 18, selected at random). All TEMPO participants had at least one parent enrolled in the GAZEL cohort (Goldberg et al. 2007). This link to parental longitudinal data, available since 1989, allows for the inclusion of parent-level factors associated with offsprings substance use, in line with a family-based approach. Due to high attrition, recruitment was extended in 2011 (Supplementary Fig. 1). TEMPO participants were surveyed in 1999, 2009, 2011, 2015, and 2018. During the COVID-19 pandemic, nine waves of data collection were carried out between March 24, 2020, one week after the beginning of the first COVID-19-related lockdown in France, and May 2021. Data were collected weekly for the first five surveys, fortnightly for the sixth and seventh surveys, and then again during the summer of 2020, as well as during the second and third lockdown and curfew periods (Supplementary Fig. 2). The TEMPO cohort included 3,411 participants, of whom 2,175 provided data on cannabis use. As the objective of this study was to examine persistent cannabis use from adolescence to adulthood, only the 622 participants who reported using cannabis at least once were included in the analysis (Fig. 1).

### Measures

All measures are prospective and self-reported, except for participants included in 2011 (*n* = 164), who answered retrospectively regarding childhood factors.

#### Outcome: cannabis use trajectories

Cannabis use frequency over the past 12 months was assessed 14 times between 1999 and 2021 (across five waves between 1999 and 2018, and nine additional waves during the COVID-19 pandemic), for participants aged 15 or older, using the question: “*Have you ever used cannabis (hash*,* marijuana*,* weed*,* joint*,* pot) in the past 12 months?*” Response options included: “*Never*”, “*1–2 times*”, “*3–5 times*”, “*6–9 times*”, “*10–19 times*”, “*20–39 times*”, and “*40 + times*”. The question, initially defined in 1999, was adapted from the *Monitoring the Future* survey (Johnston et al. 1995), and the same question was repeated in each wave to allow for comparisons across years.

For individuals who reported cannabis use at least once in the TEMPO cohort, longitudinal trajectories of cannabis consumption were modeled using Group-Based Trajectory Modeling (GBTM) (Nagin 1999, Nagin 2005; Nagin and Odgers 2010). This semiparametric method identifies and summarizes latent subgroups within a population to analyze temporal patterns and age-related changes. In the first step, models with 1, 2, 3, 4 or 5 trajectories were tested. The optimal number of trajectories was selected based on the Bayesian Information Criterion (BIC). The model with the lowest BIC absolute value was considered the best fit, provided that each trajectory included at least 10% of the sample to ensure robustness and interpretability. Compared to the Akaike Information Criterion (AIC), the BIC applies a stronger penalty for complex models. After determining the number of trajectories, all possible combinations of polynomial orders (ranging from 0 to 4) for the trajectory shapes were tested. The best-fitting trajectory shapes were selected using several a posteriori validation criteria: Average Posterior Probability (AvePP > 0.70 for each group), Odds of Correct Classification (OCC > 5 for each group), and concordance between the estimated and observed prevalence of each group (Jones et al. 2001). Each participant was assigned to the trajectory with the highest membership probability.

During the COVID-19 pandemic, survey waves were administered in close succession, resulting in minimal changes in participants’ age over time. Therefore, for the purpose of trajectory modeling, we included only one wave per year, the one with the highest reported level of cannabis use.

In accordance with Nagin’s recommendation (Nagin 2005), a sensitivity analysis was conducted among participants with at least two measures of cannabis use to validate the trajectories estimated based on participants with at least one measure.

#### Covariates of interest

All predictive factors were assessed in childhood to ensure they preceded cannabis use trajectories.

#### Sociodemographic characteristics

Participants’ sociodemographic characteristics included sex (female vs. male) and year of birth (1974–1979 vs. 1980–1995).

#### Schooling characteristics

Academic difficulties were measured using data on grade retention during primary, middle, or secondary school (1991–2011) and categorized as “No” vs. “Yes (at least one retention)”. Grade retention was modeled as ever versus never due to the low frequency of multiple retentions and potential recall error.

#### Tobacco and cannabis initiation

Age at first experimentation with tobacco and cannabis was recorded at each survey wave from 1999 onward. The earliest available information was used and dichotomized into early vs. late initiation to capture the elevated risk associated with early onset while avoiding assumptions of linearity, consistent with prior works (Breslau et al. 1993; Hamaoui et al. 2025; Volkow et al. 2021). Cut-points were based on the median age of initiation (Richmond-Rakerd et al. 2017): ≤14 vs. >14 years for tobacco, and ≤ 16 vs. >16 years for cannabis. Due to collinearity because, in France, cannabis is most often smoked with tobacco, these variables were combined into four categories: “Late or no use of tobacco and cannabis”, “Early tobacco use only”, “Early cannabis use only” and “Early use of both tobacco and cannabis”. Even if age of initiation may implicitly influence trajectory membership, the latter does not derived directly from age of initiation. Including it as an associated factor allows to test whether it impacts certain trajectories rather than others and to compare the relative weight of this factor with others. In addition, examining co-initiation with tobacco provides complementary information compared to early cannabis initiation alone, as it highlights the dynamics of trajectories and the role of a cultural and behavioral contextual factors.

#### Juvenile behavioral problems

Juvenile behavioral problems before age 17, including externalizing symptoms, were assessed using the Child Behavior Checklist (CBCL) (Achenbach 1991), completed by parents in 1991 or by participants in 1999 if they were younger than 17. Because of potential discrepancies between children’s mental health reports from parents and from the children themselves, with parents tending to underreport symptoms (Caqueo-Urizar et al. 2022; Chen et al. 2017; De Los Reyes et al. 2015), we primarily relied on child reports. The CBCL covers children aged 4 to 16, and its French translation has been adapted to this age group and its cross-cultural adaptation evaluated (Vermeersch and Fombonne 1997; Stanger et al. 1994). Items were rated on a three-point scale (0 = “*Not at all true*”; 1 = “*Sometimes or somewhat true*”; 2 = “*Very true or often true*”). Responses were summed, standardized, and dichotomized. As recommended by the authors, a standardized score above the 85th percentile classified participants as exhibiting externalizing symptoms (Achenbach and Ruffle 2000). For 2011 participants, this information was supplemented by the The Mini International Neuropsychiatric Interview (MINI) (Sheehan, 1997). The MINI has been validated among adolescents (LeBlanc et al. 2002; Sheehan et al. 2010) and in the French population (Duburcq et al. 1999; Lecrubier et al. 1997). If externalizing symptoms were reported in at least one wave, the participant was classified as “Yes (at least one)” vs. “No”.

#### Adverse childhood experiences

Adverse experiences before age 17 were reported either in the GAZEL study (when related to parents) or in the TEMPO study by parents in 1991 or by participants in 2011. These experiences included parental unemployment or financial problems, family conflict, parental stress, frequent parental absence, parental divorce, parental depression, serious parental illness or health problems, illness or death of a close family member or friend, and childhood violence (before age 15). Due to collinearity, parental stress, conflict, and frequent absence were combined into a single variable: “No” vs. “Yes (at least one).” Moreover, combine these 3 variables reflects a theory-driven construct of family dysfunction/household instability. These indicators co-occur, operate at the same ecological level, and plausibly influence substance-use trajectories through shared mechanisms (reduced parental monitoring, elevated stress reactivity, disrupted family routines) (Hughes et al. 2017).

#### Parental characteristics

Parental occupational grade was reported in the GAZEL study (1989–2015) (Goldberg et al. 2007), with the highest reported grade retained: “*Manager*”, “*Technician or administrative associate*”, or “*Manual worker or clerk*”. Parental smoking status (“*Non-smoker*”, “*Former-smoker*”, or “*Smoker*”) and parental alcohol abuse (“*No Alcohol Abuse*” or “*Alcohol Abuse*”) were based on GAZEL reports (1989–2015) and reports from children about the other parent in TEMPO (2009 or 2011).

### Statistical analysis

Chi-Square or Fisher’s exact tests were used to compare participant characteristics across cannabis trajectories. Approximately 4.2% of the covariate data were missing and were imputed using multiple imputation via Fully Conditional Specification (Buuren et al. 2006), with 20 imputations (Bodner 2008; White et al. 2011). The imputed variables were Academic difficulties, Age at first tobacco/cannabis initiation, Externalizing symptoms, Parental conflicts, stress, or frequent absence, Parental occupational grade, and Parental tobacco status. The pool function combines the 20 imputed datasets using Rubin’s rules (Rubin 1987). It calculates a weighted average of the coefficients, accounting for both within-imputation and between-imputation variability. Models were performed with and without imputations to evaluate the robustness of the findings.

Multinomial logistic regression was used to assess associations between cannabis use trajectories and early individual and family factors, with the “declining consumption” trajectory as the reference group. All variables identified a priori associated with cannabis trajectories on the basis of clinical relevance and prior literature, as well as those with *p* < 0.2 in univariable analyses were included in the multivariable model (Hosmer et al. 2013; Bursac et al. 2008; Heinze and Dunkler 2017), in order to achieve balanced adjustment without overfitting while retaining potentially important predictors. Correlations and interactions between selected variables were tested. All analyses were conducted using SAS^®^ 9.4 and R 4.1.0 (packages *nnet* (Venables and Ripley 2002), and *mice* (van Buuren and Groothuis-Oudshoorn 2011)).

## Results

**Fig. 2:**
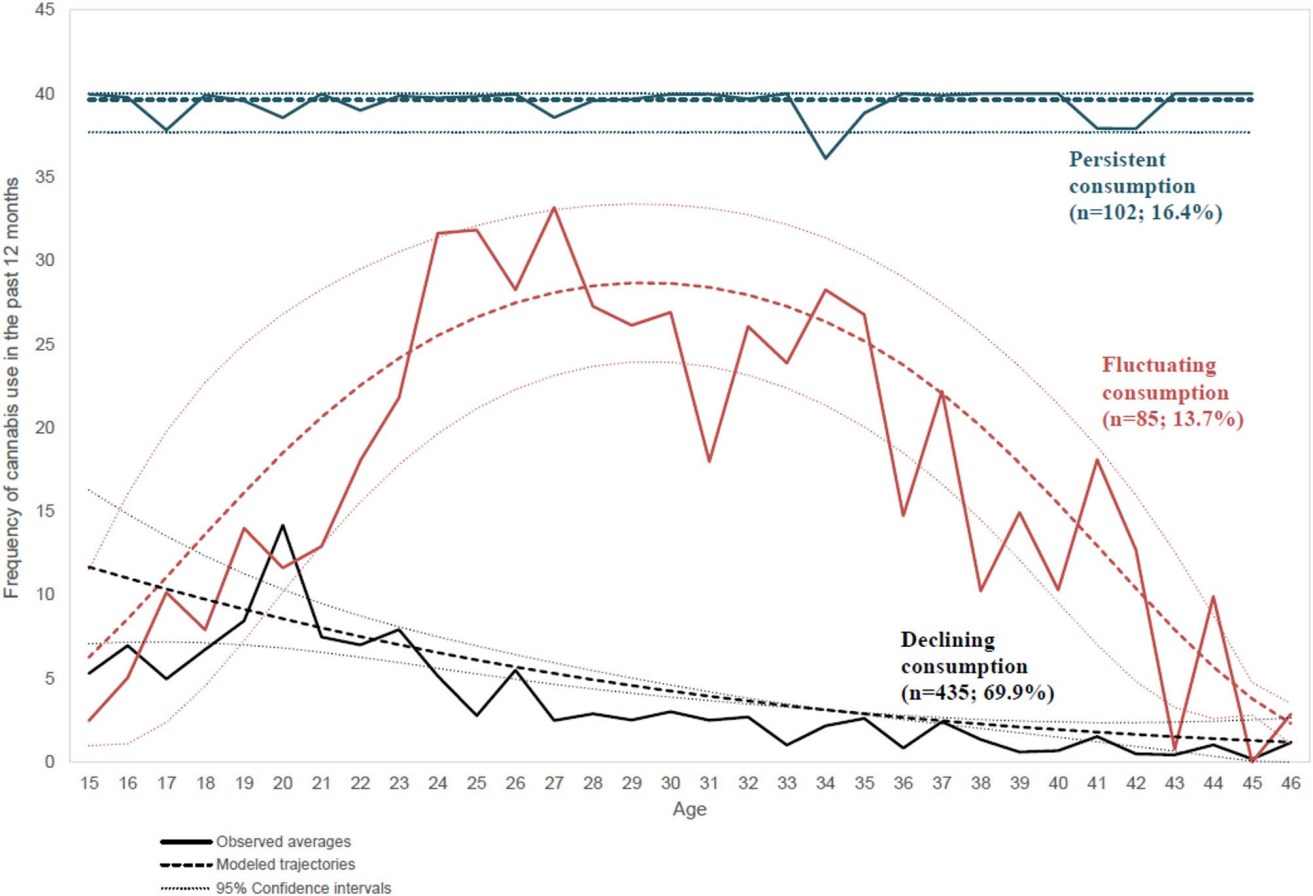
Cannabis use trajectories among TEMPO study participants (*n* = 622)

Three cannabis use trajectories were identified among study participants (Fig. 2) corresponding to a BIC=−4129: declining consumption (*n* = 435, 69.9%), fluctuating consumption, characterized by an initial increase followed by a decrease (*n* = 85, 13.7%), and persistent consumption (*n* = 102, 16.4%). The final polynomial orders were 2-2-0 for declining, fluctuating, and persistent consumption, respectively. The corresponding AvePP values were 0.94, 0.86, and 0.87. The corresponding OCC were 6.9; 37.5, and 34.3.

After identifying trajectories among individuals with at least two measures of cannabis use, the number and shape of the trajectories remained unchanged, and the distribution across groups was similar to that observed in the analysis based on individuals with at least one measure.

Table 1 presents the characteristics of the participants. On average, participants took part in 4.7 out of the 14 study waves (SD = 3.8). The fluctuating group reported the highest rates of parental conflict, stress, or frequent absence before age 17 (46.9%) compared to the other groups. Participants in the persistent consumption group, who had the highest initial levels of cannabis use, were more often male (73.5%), had experienced academic difficulties (77.0%), and were more likely to have initiated tobacco and cannabis use at an early age (42.9%).

**Table 1:** Characteristics of TEMPO participants included in the study (n=622) according to cannabis use trajectories

Variable*, n (%)*	Overall (*n* = 622)	Declining consumption (*n* = 435)	Fluctuating consumption (*n* = 85)	Persistent consumption (*n* = 102)	*p*-value^1^
*Sociodemographic variables*					
Sex					**<0.001**
Female	320 (51.4%)	257 (59.1%)	36 (42.4%)	27 (26.5%)	
Male	302 (48.6%)	178 (40.9%)	49 (57.6%)	75 (73.5%)	
Year of birth					0.060
1974-1979	255 (41.0%)	173 (39.8%)	30 (35.3%)	52 (51.0%)	
1980-1995	367 (59.0%)	262 (60.2%)	55 (64.7%)	50 (49.0%)	
*Schooling characteristics*					
Academic difficulties	344 (56.3%)	223 (52.1%)	44 (53.0%)	77 (77.0%)	**<0.001**
*Tobacco and cannabis initiation*					
Age at first experimentation of tobacco and cannabis					**0.006**
Late or no tobacco and cannabis	211 (39.4%)	163 (42.7%)	30 (39.5%)	18 (23.4%)	
Early tobacco	83 (15.5%)	66 (17.3%)	8 (10.5%)	9 (11.7%)	
Early cannabis	86 (16.1%)	55 (14.4%)	14 (18.4%)	17 (22.1%)	
Early tobacco and cannabis	155 (29.0%)	98 (25.7%)	24 (31.6%)	33 (42.9%)	
*Juvenile behavioral problems (before age 17)*					
Externalizing symptoms	123 (21.1%)	81 (19.8%)	22 (27.2%)	20 (21.7%)	0.324
*Adverse childhood experiences (before age 17)*					
Parental unemployment or financial problems^2^	38 (8.6%)	24 (7.5%)	6 (10.9%)	8 (11.9%)	0.334
Parental conflict, stress or frequent absence^2,3^	203 (33.8%)	132 (31.4%)	38 (46.9%)	33 (33.3%)	**0.025**
Parental divorce^4^	50 (8.1%)	33 (7.6%)	8 (9.4%)	9 (8.8%)	0.818
Parental depression^4^	91 (14.7%)	62 (14.3%)	10 (11.8%)	19 (18.6%)	0.386
A serious illness or health problem^2^	47 (10.6%)	33 (10.3%)	8 (14.5%)	6 (9.0%)	0.569
The illness of a close family member or friend^2^	114 (25.7%)	75 (23.4%)	16 (29.1%)	23 (33.8%)	0.165
Death of a close family member or friend^2^	59 (13.3%)	46 (14.3%)	6 (10.9%)	7 (10.4%)	0.595
Violence experienced (before age 15)^3^	157 (48.0%)	110 (47.4%)	25 (46.3%)	22 (53.7%)	0.733
*Parental characteristics*					
Parental occupational grade					0.104
Manager	382 (61.5%)	265 (61.1%)	62 (72.9%)	55 (53.9%)	
Technician or administrative associate	208 (33.5%)	147 (33.9%)	21 (24.7%)	40 (39.2%)	
Manual worker or clerk	31 (5.0%)	22 (5.1%)	2 (2.4%)	7 (6.9%)	
Parental tobacco status					0.067
Non-smoker	200 (32.2%)	152 (35.0%)	19 (22.4%)	29 (28.4%)	
Former-smoker	203 (32.7%)	141 (32.5%)	26 (30.6%)	36 (35.3%)	
Smoker	218 (35.1%)	141 (32.5%)	40 (47.1%)	37 (36.3%)	
Parental alcohol abuse					0.652
Non-Alcohol Abuse	411 (66.2%)	292 (67.3%)	55 (64.7%)	64 (62.7%)	
Alcohol Abuse	210 (33.8%)	142 (32.7%)	30 (35.3%)	38 (37.3%)	

^1^ *p*-value obtained by Pearson's Chi-squared test or Fisher's exact test (for categorical variables with any expected cell count below 5)

^2^ Reported by parents in 1991

^3^ Reported by participants in 2011

^4^ Reported by parents in the GAZEL study (1989-2015)

Table 2 presents the results of the multivariable multinomial logistic regression, adjusted for sex, year of birth, academic difficulties in primary, middle, or secondary school, age at first experimentation with tobacco and cannabis, externalizing symptoms before age 17, parental conflict, stress, or frequent absence before age 17, parental socioeconomic status, and parental smoking status. Compared to the declining consumption group, fluctuating users had increased odds of being male (OR = 2.15, 95%CI = 1.31–3.54), to have experienced parental conflict, stress, or frequent absence before age 17 (OR = 1.93, 95%CI = 1.15–3.23), and to have parents who smoked (OR = 2.18, 95%CI = 1.18–4.02). Persistent users had increased odds of being male (OR = 3.66, 95%CI = 2.19–6.09), to have experienced academic difficulties (OR = 2.47, 95%CI = 1.45–4.22), and to have initiated early use of both tobacco and cannabis (OR = 3.07, 95%CI = 1.57–6.02) or early cannabis use alone (OR = 2.31, 95%CI = 1.11–4.79).

**Table 2:** Factors associated with cannabis use trajectories by multinomial logistic regression (TEMPO cohort study, 1999–2021, France, *n* = 622; odds-ratio (OR) and 95% confidence interval (95% CI))

	Univariable model^2^				Multivariable imputed^1^ model^2^			
	Fluctuating consumption (*n* = 85)		Persistent consumption (*n* = 102)		Fluctuating consumption (*n* = 85)		Persistent consumption (*n* = 102)	
	OR [95% CI]	*p*-value	OR [95% CI]	*p*-value	OR [95% CI]	*p*-value	OR [95% CI]	*p*-value
Sex								
Female	reference		reference		reference		reference	
Male	1.97 [1.23–3.15]	**0.005**	4.01 [2.48–6.48]	**< 0.001**	2.15 [1.31–3.54]	**0.003**	3.66 [2.19–6.01]	**< 0.001**
Year of birth								
1974–1979	reference		reference		reference		reference	
1980–1995	1.21 [0.75–1.97]	0.440	0.63 [0.41–0.98]	**0.040**	1.21 [0.72–2.04]	0.467	0.63 [0.38–1.02]	0.061
Academic difficulties^3^								
No	reference		reference		reference		reference	
Yes (at least one^4^)	1.04 [0.65–1.66]	0.879	3.08 [1.86–5.09]	**< 0.001**	0.89 [0.54–1.46]	0.633	2.47 [1.45–4.22]	**0.001**
Age at first experimentation of tobacco and cannabis								
Late or no tobacco and cannabis	reference		reference		reference		reference	
Early tobacco	0.66 [0.29–1.51]	0.324	1.23 [0.53–2.89]	0.626	0.73 [0.31–1.74]	0.479	1.32 [0.56–3.14]	0.522
Early cannabis	1.38 [0.68–2.80]	0.367	2.80 [1.35–5.81]	**0.006**	1.20 [0.58–2.46]	0.622	2.31 [1.11–4.79]	**0.025**
Early tobacco and cannabis	1.33 [0.74–2.41]	0.345	3.05 [1.63–5.71]	**< 0.001**	1.12 [0.59–2.13]	0.722	3.07 [1.57–6.02]	**0.001**
Externalizing symptoms^3^								
No	reference		reference		reference		reference	
Yes (at least one^4^)	1.51 [0.88–2.62]	0.137	1.13 [0.65–1.96]	0.668	1.26 [0.70–2.24]	0.441	0.82 [0.44–1.52]	0.525
Parental conflicts, stress or frequent absence^3^								
No	reference		reference		reference		reference	
Yes (at least one^4^)	1.93 [1.19–3.13]	**0.007**	1.09 [0.69–1.74]	0.704	1.93 [1.15–3.23]	**0.013**	1.07 [0.64–1.80]	0.788
Parental occupational grade								
Manager	reference		reference		reference		reference	
Technician or administrative associate	0.61 [0.36–1.04]	0.070	1.31 [0.83–2.07]	0.243	0.61 [0.35–1.05]	0.0764	1.28 [0.78–2.08]	0.4322
Manual worker or clerk	0.39 [0.09–1.70]	0.209	1.53 [0.62–3.77]	0.351	0.40 [0.09–1.78]	0.226	1.60 [0.58–4.38]	0.364
Parental tobacco status								
Non-smoker	reference		reference		reference		reference	
Former-smoker	1.48 [0.78–2.78]	0.230	1.34 [0.78–2.30]	0.290	1.57 [0.82–3.02]	0.174	1.18 [0.66–2.13]	0.575
Smoker	2.27 [1.26–4.10]	**0.007**	1.38 [0.80–2.35]	0.245	2.18 [1.18–4.02]	**0.013**	1.22 [0.69–2.18]	0.493

^1^ Imputed with MICE

^2^ Each trajectory of cannabis use is compared to the reference trajectory: Declining consumption (*n* = 435)

^3^ Before age 17

^4^ Notified in at least one wave before the age of 17

## Discussion

This longitudinal study contributes to the existing, albeit limited, body of literature on cannabis use patterns from adolescence to adulthood. In a large, heterogeneous cohort of young people followed prospectively, we found that among adolescent cannabis users, approximately 70% reduced their use over time, 14% reported fluctuating use, and 16% exhibited persistent consumption. Early-life factors, including academic difficulties, and early cannabis/tobacco use, were predictive of persistent use, whereas adverse childhood experiences such as parental conflicts, stress, or frequent absence were predictive of fluctuating use, suggesting that these factors exert greater influence during adolescence and early adulthood than during adulthood. In a context where adult cannabis use has increased over time, contrasting with declining trends among adolescents, our study suggests that early-life factors influence long-term risk and should be targeted by interventions aiming to prevent adverse outcomes related to cannabis use.

The declining consumption group, representing the majority of the sample, aligns with findings showing that approximately half of cannabis users spontaneously cease use in their twenties (Terry-McElrath et al. 2017; von Sydow et al. 2001). In contrast, persistent consumption may be linked to higher initial levels of use. This is consistent with previous research (Kosty et al. 2017), which identified three similar cannabis trajectory classes, referring to the fluctuating group as “the maturing out” group. This concept, also discussed by Marmet et al., describes how CUDs often emerge in adolescence but mature out (decrease or cease) as individuals age (Marmet et al. 2021). Similarly, Passarotti et al. identified three marijuana use trajectories (low, medium, and high users), including an escalating use group (Passarotti et al. 2015). As participants were followed from age 14–15 for six years, this upward trajectory is not incompatible with the fluctuating trajectory observed in our study or in the study of Terry-McElrath et al. (Terry-McElrath et al. 2017) which shows a decrease in consumption after the age of 27.

The fluctuating trajectory, with a decline in cannabis use from ages 27–30, could be explained by key life events such as entering the workforce, couple formation, pregnancy in women, and parenthood, These factors may contribute to decisions to reduce cannabis consumption due to the risk of cannabis-related impairment in the workplace and increased likelihood of accidents (Wolfrom & Victor, 2020), the negative impact of discrepant cannabis use on couple functioning (Testa et al. 2018), including intimate partner violence (Cunradi et al. 2015), the risk for the baby during pregnancy (Azubuike et al. 2025; Baranger et al. 2024), and the desire to provide exemplary parenting (Rezag Bara et al. 2023).

We found that men had higher odds of belonging to the persistent consumption trajectory and, to a lesser extent, the fluctuating trajectory. Even though the gender gap in cannabis use has narrowed in recent years (Chapman et al. 2017; Goodwin and Silverman 2024; Le Nézet et al., 2020), one possible explanation is that men generally use substances more frequently than women (Carliner et al. 2017; Coffey et al. 2003; Cutler et al. 2016). In France, more than one in two men (54.8%) have experimented with cannabis compared to fewer than four in ten women (37.7%) (Le Nézet et al., 2020), placing men at higher risk for sustained heavy use. This aligns with prior research suggesting gender-related risk mechanisms, as men are more likely to be diagnosed with CUD over their lifetime (Kosty et al. 2017; Farmer et al. 2015).

Our findings also showed that individuals born after 1980 were 37% less likely to engage in heavy cannabis use. This may reflect evolving societal attitudes toward cannabis, including political policies and actions, as well as prevention programs (Yu at al., 2020). Another possible explanation is the recent diversification of psychoactive substances (e.g., MDMA, cocaine, recreational alcohol, video games, etc.), which may have led to less intensive cannabis use despite frequent experimentation. The European Union Drugs Agency (EUDA) has observed that younger generations are more likely to use cannabis occasionally or in combination with other substances, rather than intensively and regularly (European Union Drugs Agency 2021). Recent data indicate a decline in cannabis use among younger populations in France, suggesting shifting trends (Observatoire Français des Drogues et des Tendances addictives 2023). Additionally, early initiation of tobacco and cannabis was significantly associated with persistent use. Specifically, individuals who initiated both substances early, or cannabis alone, were approximately three times more likely to follow a persistent use trajectory. This finding is consistent with extensive literature showing that early cannabis initiation increases lifetime risk of developing CUD (Copeland et al. 2014; Kalant 2004). Ellickson et al. further reported that early heavy users experience the most adverse outcomes, including poorer health and greater involvement with hard drugs (Ellickson et al. 2004).

Academic difficulties were also associated with persistent cannabis use, with affected individuals being 2.35 times more likely to belong to this trajectory. This supports earlier studies linking high cannabis use to lower educational attainment by age 25 (Fergusson and Boden 2008), higher dropout rates (Fergusson and Horwood 1997), poor academic performance, lower school satisfaction, and negative attitudes toward education (Lynskey and Hall 2000). Additionally, early cannabis users have been found to attain lower levels of education than non-users (Melchior et al. 2017). Several hypotheses have been proposed to explain these associations, including the cannabis-related “amotivation syndrome”, cognitive impairments, and social factors influencing initiation (Lynskey and Hall 2000).

Furthermore, individuals who experienced parental conflict, stress, or frequent absence before age 17 were twice as likely to follow a fluctuating cannabis use trajectory. Prior studies have shown that early exposure to stress, conflict, and negative emotions within the family environment increases the risk of cannabis initiation in adolescence (Madu and Matla 2003; Vakalahi 2001). Poor parent-child communication and lower family cohesion have also been identified as predictors of high and decreasing use patterns (Cardenas et al. 2022). Similarly, Skeer et al. found that adolescents exposed to family conflict were 1.23 times more likely to engage in substance use (Skeer et al. 2009). In terms of substance use trajectories, Brook et al. reported that maternal risk factors, such as smoking, increased the likelihood of substance use, whereas maternal affection was associated with lower use (Brook et al. 2012).

Participants with smoking parents had higher odds of belonging to the fluctuating consumption trajectory compared to those with non-smoking parents. Although smoking prevalence among adults in France decreased from 30.0% in 2000 to 25.3% in 2021 (Obradovic et al. 2023), parents often underestimate the risk of their children adopting similar behaviors. One study found that 59% of parents believed their children would not initiate cannabis use, and 77% thought the same for tobacco (Chadi et al. 2020). Conversely, a qualitative study reported that most medical marijuana patients did not want their children to use cannabis (Rezag Bara et al. 2023; Thurstone et al. 2013). However, research suggests that parental substance use not only directly influences children’s behavior but also shapes their attitudes toward substances (Wilkinson et al. 2008).

### Limitations

This study has several limitations. First, the generalizability of the findings is limited, as the TEMPO cohort include participants with higher socioeconomic status and income than the general population (Mary-Krause et al. 2021), given that their parents were part of the GAZEL study (Goldberg et al. 2007). Nevertheless, TEMPO participants remain diverse in terms of geography and socioeconomic background, allowing for meaningful analysis of associations between risk factors and cannabis use trajectories, although this may lead to underestimating the strength of some associations. Additionally, the identification of three distinct trajectories suggests sufficient heterogeneity in the sample. Second, data were collected via self-reported questionnaires, which may introduce social desirability bias and underreporting of substance use (Kilian et al. 2021). However, self-reports remain widely used in substance use research, and previous studies have validated their reliability (Curran et al. 2019; Skelton et al. 2022). Additionally, confidential questionnaires yield more accurate reports than face-to-face interviews (Mayet et al. 2013; Moskowitz 2004). Third, the categories used to assess cannabis use were not formally validated, but they were adapted from a well-known U.S. national survey (*Monitoring the Future*, Johnston et al. 1995) and have been employed in several cohort studies and national surveys (Manrique-Garcia et al. 2012; McGee et al. 2000; ICPSR,^ 2025^). Although cannabis use in the past 12 months is commonly used in research, studies often rely on a binary classification (yes vs. no use, i.e., at least once) due to limited statistical power in more granular categories (Afifi et al. 2024; Hoeg et al. 2024; Manrique-Garcia et al. 2012; McGee et al. 2000; Schöllner et al., 2025). The use of more detailed categories in our study allows for greater specificity. Forth, participants provided data at an average of 5 out of the 14 time points. Nevertheless, most individuals participated during their childhood and adolescence, resulting in limited missing data on potential predictive factors. Furthermore, a strength of GBTM is its ability to model trajectories even when only a few follow-up points are available. Therefore, although participants did not respond to all waves, the findings remain informative and valuable. Fifth, previous literature has highlighted discrepancies between children’s mental health as reported by parents and by children themselves. Children tend to report more internalizing and externalizing symptoms than their parents (Caqueo-Urizar et al. 2022; Chen et al. 2017; De Los Reyes et al. 2015). Such discrepancies could lead to an underestimation of the ORs corresponding to internalizing and externalizing symptoms when parent reports are used. In our study, as the majority of participants were assessed in 1991 and 1999, we primarily relied on child reports, which reduced this potential underestimation. Finally, an important missing factor in this study is parental history of illicit drug use during participants’ childhood or adolescence (Boden et al. 2020). Unfortunately, parental use of cannabis or other illicit drug use is not reported in the GAZEL cohort. Research has shown that parental cannabis use during adolescence increases the risk of early initiation among offspring (Tiberio et al. 2020), suggesting an intergenerational dynamic. Nonetheless, a qualitative analysis of TEMPO participants indicated that parents who had used cannabis often framed it negatively, sometimes even demonizing its use (Rezag Bara et al. 2023).

### Strengths

Despite its limitations, this study presents several strengths. First, the TEMPO cohort is an ongoing community-based longitudinal cohort enabling the follow-up of 622 cannabis users from adolescence into adulthood over a 30-year period (1991–2021). This allowed for robust longitudinal tracking of cannabis use trajectories. Second, early-life and family characteristics were collected independently of participants’ substance use reporting, minimizing recall bias. Prospective data included information on various childhood factors, including externalizing symptoms before age 17, academic difficulties, and parental characteristics such as divorce, depression, and substance use. Third, the familial design of the study, with parallel data from both parents and children, enabled a thorough examination of familial influences. Additionally, the use of GBTM method allowed us to identify distinct patterns of cannabis use while accounting for multiple influencing factors (Caldeira et al. 2012). Finally, to our knowledge, this is one of the only large-scale French studies to investigate cannabis use trajectories with such an extended follow-up.

## Conclusions

This study identifies diverse cannabis use trajectories among individuals followed from adolescence to adulthood over three decades in France, and highlights several associated early-life and familial factors. Our findings carry important public health implications, underscoring the need for early intervention among young people and support strategies targeting at-risk youth, particularly in contexts where cannabis remains illegal yet widely used. Given the potential negative consequences of cannabis use on physical, mental, and social health, effective policies are needed to reduce its consumption. Crucially, such policies should address early-life risk factors to maximize their preventive impact. Prevention may take different forms, at both individual and family level. Examples include school-based prevention programs, accurate information about the risks associated with use, family support services, and early screening for behavioral problems. Motivational interviewing and person-centered therapies should also be systematically offered to facilitate cessation. Furthermore, raising awareness among caregivers is essential so they can recognize adolescents who have experienced adverse life events and help prevent subsequent psychoactive substance use.

## Supplementary Information

Supplementary Material 1: Figure 1. Timeline of TEMPO and TEMPO COVID-19 data collection and number of participants from 1991 to 2021.

Supplementary Material 2.

## Data Availability

Due to the personal nature of the questions asked in this study, research participants were guaranteed that the raw data would remain confidential. Upon reasonable request and in accordance with General Data Protection Regulation (GDPR) standards, data may be accessed by sending an email to cohort.tempo@inserm.fr . Anonymized data can only be shared after explicit approval of the French national committee for data protection for approval (*Commission Nationale de l’Informatique et des Libertés, CNIL*).
