# Supplementary figures and images for: Childhood events as factors in continued cannabis use in adulthood: a longitudinal study of a 30-year follow-up cohort
Source: J Cannabis Res. 2025 Nov 12;7:89. doi: 10.1186/s42238-025-00345-0 (PMC12613728; doi:10.1186/s42238-025-00345-0)

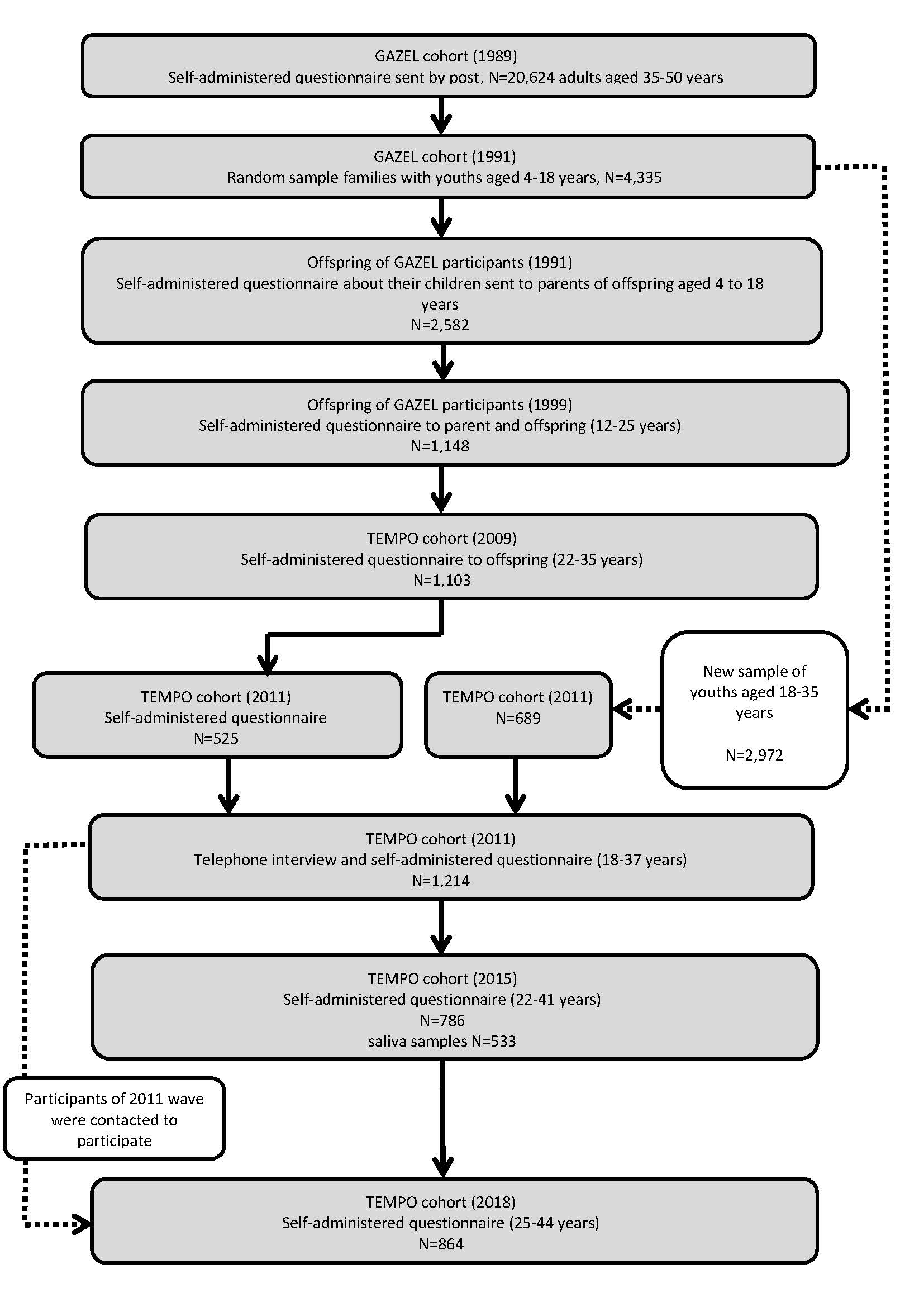

Supplement: Supplementary file 1 — Supplementary Material 1: Figure 1. Timeline of TEMPO and TEMPO COVID-19 data collection and number of participants from 1991 to 2021. [file 42238_2025_345_MOESM1_ESM.jpg]

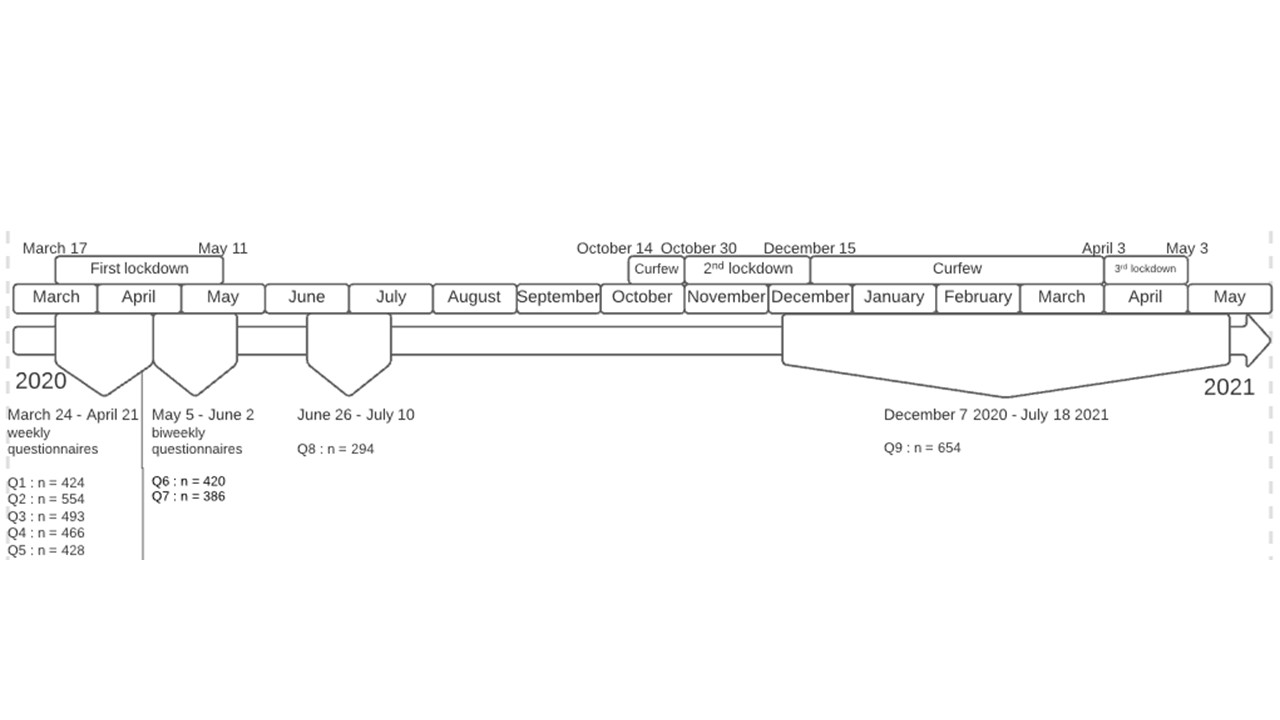

Supplement: Supplementary file 2 — Supplementary Material 2. [file 42238_2025_345_MOESM2_ESM.jpg]
